# Aridity influences the recovery of vegetation and shrubland birds after wildfire

**DOI:** 10.1371/journal.pone.0173599

**Published:** 2017-03-29

**Authors:** Roger Puig-Gironès, Lluís Brotons, Pere Pons

**Affiliations:** 1 Departament de Ciències Ambientals, Universitat de Girona, Girona, Catalonia, Spain; 2 Forest Sciences Centre of Catalonia (CEMFOR-CTFC), Solsona, Catalonia, Spain; 3 Centre de Recerca Ecològica i Aplicacions Forestals (CREAF), Autonomous University of Barcelona, Bellaterra, Catalonia, Spain; USDA-ARS Fort Keogh Livestock and Range Research Laboratory, UNITED STATES

## Abstract

Wildfires play a determining role in the composition and structure of many plant and animal communities. On the other hand, climate change is considered to be a major driver of current and future fire regime changes. Despite increases in drought in many areas of the world, the effects of aridity on post-fire colonization by animals have been rarely addressed. This study aims to analyse how a regional aridity gradient affects post-fire recovery of vegetation, bird species richness and the numbers of four early to middle-successional warbler species associated with the shrub cover. The database contains bird relative abundance and environmental variables from 3072 censuses in 695 transects located in 70 recently burnt areas (1 to 11 years after wildfire) in Catalonia (Spain), which were sampled between 2006 and 2013. Generalized linear mixed models (GLMMs) showed that plant cover was affected by time since fire, aridity and forest management. However, only the highest vegetation height layer (>100 cm) recovered slower in arid areas after fire. Time since fire positively influenced bird species richness and the relative abundance of the four focal species. The post-fire recovery of Melodious (*Hippolais polyglotta*) and Subalpine warblers (*Sylvia cantillans*) was hampered by aridity. Although this was not demonstrated for Dartford (*S*. *undata)* and Sardinian warblers (*S*. *melanocephala)*, their occurrence was low in the driest areas during the first three years after fire. Overall, this study suggests that future increases in aridity can affect plant regeneration after fire and slow down the recovery of animal populations that depend on understorey and shrublands. Given the recently highlighted increases in aridity and fire frequency in Mediterranean-climate regions, improved knowledge on how aridity affects ecological succession is especially necessary.

## Introduction

Summer droughts are probably the most conspicuous feature of the Mediterranean climate. Due to water stress, occurring when evapotranspiration (i.e. the sum of water lost in evaporation and transpiration) is high during a long period [1], plant development is severely hindered [2], and drought tolerant shrublands and sclerophyllous forests are the characteristic habitats [3]. Further, wildfires are major disturbances in these regions, playing a decisive role in the dynamics and structure of plant and animal communities [4]. Wildfire occurrence is expected to increase due to global climate change in Mediterranean regions, affecting larger areas and burning with greater intensity [5]. The recurrence of fire is highest in areas with semi-humid Mediterranean climates and lower in both the driest and the wettest areas [6]. Geographical dryness gradients therefore determine the vegetation structure and influence wildfire risk [7].

Most Mediterranean habitats can recover their original structure and composition following a wildfire [8], due to the resilience of the plant community [9], a result of the combined responses by different plant functional types [10]. However, mid-successional Mediterranean-type shrublands can also remain unwooded [11] because of several factors, including (1) adverse environments (arising from geology, topography or soil limitation), (2) intensive land use trajectories, and (3) high recurrence of disturbances, that may not allow development of tree cover. Among the environmental factors limiting post-fire regeneration, water availability may lead to a differential response to stress conditions [12] and induce changes in the proportion of plant functional types. Although obligate seeders (i.e. plant species that regenerate only from the seed bank) have a higher drought tolerance than resprouters (post-fire sprouting thanks to dormant buds [10]), the resilience, height and biomass of the vegetation as a whole depends on water availability during the early years after fire [13]. Moreover, cover combustion by fire leads to a higher incidence of solar radiation on the ground, increasing evapotranspiration and, consequently, increasing the hydric stress that affects the establishment of vegetation [14].

Although aridity gradients may affect continental patterns of bird richness [15], water limitation does not usually affect insectivorous birds directly, since their water needs are met by food [16]. However, the negative effects of aridity on plant regeneration [17] can indirectly influence their occurrence and abundance after fire due to their well-known dependence on vegetation structure [18]. The association of birds with the structure of early to middle-successional habitats that could be affected by aridity, can best be explored using species strongly dependent on low plant strata, such as many warblers. Mediterranean warblers of the genera *Sylvia* and *Hippolais* are shrub-dwelling insectivorous songbirds, occupying early to middle-successional habitats [19] and have been chosen as our focal species. Mediterranean warblers are affected by wildfires in the short term, because they require shrubs to forage and breed [20]. Nevertheless, the post-fire recovery of the shrub cover benefits them in the mid-term [21]. In absence of further fires, tree development degrades the quality of habitat for most warbler species in the longer term [19].

In addition to aridity and plant regeneration, fire characteristics and management practices may affect Mediterranean warblers occurrence after a fire. Wildfire size, affects recolonization and species composition [22]. For example, large burnt areas more likely offer suitable sites for species to settle, once the shrub cover has regenerated. The original habitat that was burnt also constrains the post-fire bird community [23], because it affects the remaining habitat structure as well as plant regeneration, and modulates the probability of bird species occurrence before (and after) the wildfire. The presence of unburnt or lightly burnt vegetation patches, is related to the fire severity and to fire extinction efforts. These unburnt patches act as refuges for some bird species in burnt areas [24], before the recovery of an appropriate habitat structure. On the other hand, salvage logging reduces the density of snags and affects the quantity of wood debris. Snags and fallen dead branches remaining after timber harvesting, affect Mediterranean warblers abundance after wildfire [25, 26]. These factors should be taken into account in postfire bird studies.

Research on the interaction of the aridity gradient, wildfires and fauna responses indicates that there can be a consistent species richness response to fire-mediated landscape complexity across a rainfall gradient [27–29]. Our aim here is to assess the effect of aridity via constraints on vegetation recovery on the abundances of four early to middle-successional species of Mediterranean warblers during the first eleven years after a wildfire. For this purpose, we predicted that: (1) vegetation recovery after fire will be quicker in wetter than in drier areas; (2) warbler abundances will peak earlier in wetter areas compared to drier ones following their more rapid post-fire recovery of shrub cover; and (3) plant cover and bird occurrence after a fire will also be affected by fire characteristics and management practices.

## Methods

### Study region

**Fig 1:**
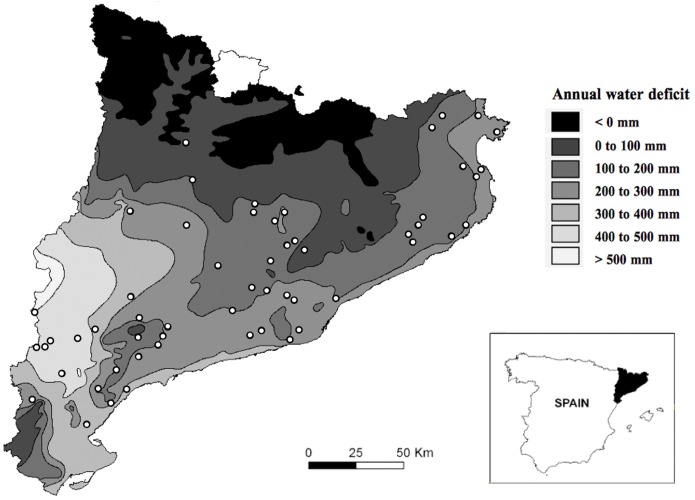
Distribution of the burnt areas and regional categories of water deficit in Catalonia. Map of Catalonia showing the locations of the 70 areas larger than 50 ha burnt by wildfires between 2000 and 2013 which were sampled in this study (modified from Zozaya et al., [34]), distributed among seven categories of annual regional water deficit (WD_A_ [35, 36]; see text for details).

The study was conducted in Catalonia (NE Iberian Peninsula), a region of some 32100 km^2^ with high environmental heterogeneity due to sharp climatic and geological gradients. Most of the study area has a Mediterranean climate, with winter precipitation and summer drought [5]. The mean annual rainfall ranges from 350 mm in the southwest to 1200 mm in the Pyrenees and aridity increases from north to south due to latitude and topography and from east to west due to continentality. Water deficit is a main environmental stressor in this area (Fig 1), because when high, the water needs of the plants are less likely to be met. Here water deficit refers to cases when the evaporative demand exceeds available soil moisture [30]. We calculated water deficit (S1 Appendix), and used it as a measure of aridity. Both terms are used hereafter interchangeably. The presence of humans since pre-historical times has led to large-scale changes in plant species composition and the distribution of dominant species throughout history. Land cover in Catalonia presently consists of forests (31%, encompassing 3/5 of conifer forests and 2/5 of forests dominated by sclerophyllous and deciduous tree species), evergreen shrublands (29%) and agricultural land (33%; that includes crops and pastures) [23, 31]. Fire is a major landscape driver in the region, with about 25% of the wild land area (i.e. non-agricultural, non-urban land) having been burnt between 1975 and 2010 [32]. In fact, land abandonment affecting the Northern Mediterranean basin has led to extensive woody plant encroachment and to a greater extent of the area being affected by wildfires in recent years. Furthermore, recent fires, under more extreme meteorological conditions promoted by global warming, have burnt with unprecedented intensity [33]. Most burnt forests are salvage logged and the resulting wood debris may be completely removed, left scattered on the ground or piled up [26].

### Sampling design

We studied vegetation and birds in 70 wild land areas ranging in size from 51 to 6647 ha that were affected by wildfires between 2000 and 2013 and distributed throughout Catalonia over an extensive water deficit gradient (Fig 1). The majority (70%) of burnt areas have moderate water deficit indexes, and are mostly located in lowlands or mountains with a Mediterranean climate. No specific permission was required for this study because transects were run on public paths (Catalan and Spanish laws guarantee the public use of paths that traverse private land), we did not capture animals and neither did we interfere with their usual behaviour.

Our database includes the results of 3071 bird censuses and measures of environmental variables in the burnt areas. With the help of a GPS device, we established transects in burnt forests and shrublands [37], each approximately 500 m in length and 200 m wide, in which environmental variables were measured and birds were censused. The number of transects depended on the area of the fire, ranging from 2 to 41 transects per burnt area (Table 1). Each survey of a transect lasted 15 minutes, divided into three 5-minute periods covering around 165m in length per period. Environmental variables were estimated at two semi-circular areas (hereafter sampling stations) of approximately 1000 m^2^ each, located at both ends of the transect. Birds were counted, when heard or seen, and were allocated into one of three distance bands (0–25 m, 25–50 m, 50–100 m). Surveys were conducted once every breeding season (from 10^th^ May to 15^th^ June) in good weather conditions (i.e. without rainfall or strong wind) during the first three hours after sunrise by experienced ornithologists walking at a speed of about 2 km/h [37]. Before the start of every sampling season, the estimation of vegetation structure, management variables (the salvage logging of burnt forests results in changes in the density of snags and branches on the ground, either scattered or piled up) and fire characteristics was standardized among the observers. The minimum distance between two transects was 150 m, and the minimum distance between transects and fire edge was 50 m. The criteria for establishing the number of transects and the sampling protocol are fully explained in Zozaya et al. [34].

**Table 1:** Summary of the sampling design according to the size range of burnt areas.

Size of burnt area (ha)	Number of transects per burnt area	Number of burnt areas
50 to 250	2 to 11	42
250 to 500	5 to 15	11
500 to 1000	15 to 22	7
More than 1000	20 to 41	10

### Vegetation and bird data

The percentage of foliage cover, an indicator of plant recovery, was estimated for three virtual vegetation height layers: lower (0–25 cm), intermediate (25–100 cm) and upper (>100 cm), by comparison with a reference chart [38] at the two sampling stations per transect. We then used the mean of the two values per transect. The number of birds counted per transect was used as a measure of species relative abundance. The minority of birds (<1%) counted on unburnt surrounding area were excluded from analyses. We studied the total species richness (i.e. the number of bird species per transect) and, more specifically, the numbers of four Mediterranean species of *Sylvia* and *Hippolais* warblers. We chose these species because they are early to middle-successional birds that disappear shortly after severe fires, then recolonize, showing unimodal abundance trends that peak a few years after the fire [39]. They can therefore be used as indicators of vegetation structure and allow comparisons to be made of the rate of species recolonization and maximum abundance after fire along a water deficit gradient.

Our four focal species are shrub-dwelling insectivorous songbirds widely distributed in the Mediterranean-climate areas of Catalonia [40]. *Sylvia* warblers are closely related to each other [41], with several species occurring in sympatry and occupying similar ecological niches [42]. The Melodious Warbler, *Hippolais polyglotta*, and the Subalpine Warbler, *Sylvia cantillans* are summer visitors that overwinter in Africa. The first species tends to occupy blackberry brambles and lush shrublands on humid soils, whereas the second one requires tall shrublands and shrublands with trees. The Sardinian Warbler, *Sylvia melanocephala*, is a common resident species in a variety of habitats of Mediterranean climate with mild winters. Finally, the Dartford Warbler, *Sylvia undata*, is a shrubland specialist, currently classified as *near threatened* [43], which mostly inhabits vegetation below 1 m in height, unlike the other three species which tend to favour higher strata [20]. The study of the post-fire population dynamics and their relationship with aridity factors (other than local changes in habitat) after fire is especially relevant for *S*. *undata*, because its populations have undergone a recent decline in Spain [44].

### Environmental variables

Water deficit (WD), our indicator of aridity, was calculated for each transect using potential evapotranspiration (PET) and real evapotranspiration (ETr) from the Digital Climate Atlas for the Iberian Peninsula [45]; a 180-m resolution digital elevation model using data from meteorological stations (one station/64km^2^). We used the equation established by Thornthwaite [46]: *WD* = *PET* – *ETr* (see S1 Appendix for more details on this equation), that gave values of between 538.3 and 0 mm for transects (WD_T_). In addition, every entire burnt area was assigned to its regional water deficit category (WD_A_; Fig 1) found in the water deficit map of Catalonia [35, 36]: 1 (WD = 0 mm), 2 (0 mm < WD ≤ 100 mm), 3 (100 mm < WD ≤ 200 mm), 4 (200 mm < WD ≤ 300 mm), 5 (300 mm < WD ≤ 400 mm), 6 (400 mm < WD ≤ 500 mm) and 7 (WD > 500 mm). These categories were used as a Random factor in the analyses (see under *Data analyses*). Time since fire was measured as the number of years that had elapsed since the fire (first spring = 1, and so on), and ranged from 1 to 11.

Because of the well-known relationship between birds and habitat structure, we took into account the management practices and fire characteristics that may affect vegetation cover and bird occurrence after a fire. With this aim in mind, we included the logging of burnt trees, the presence of scattered branches and the presence of piled branches, as management variables in our models. We also included the presence of unburnt vegetation patches, the type of pre-fire habitat and the size of the burnt area as predictor variables of the fire characteristics. Salvage logging produces a drastic habitat change a short time after fire and this was taken into account with an ordinal variable with values for each sampling station area ranging from 0 (completely unlogged) to 2 (completely logged). A logging variable was then derived by adding together the values obtained from two sampling stations per transect, resulting in a value between 0 and 4. The presence of scattered and of piled branches was recorded with values in each sampling station ranging from 0 (absent) to 2 (abundant). A plant debris variable was then derived by adding together the values of both variables (scattered and piled) from two sampling stations per transect, resulting in a value between 0 and 8. Unburnt or lightly burnt patches may facilitate the occurrence of several bird species in burnt areas, acting as habitat islands. The presence of these patches throughout the transects was measured by an ordinal variable with values ranging from 0 (the area surveyed along the transect had been completely burnt by severe fire) to 4 (many unburnt patches remained throughout the transect area). We also considered the categorical variable ‘type of pre-fire habitat’ in the area surveyed along the transect: shrubland, pine forest or oak forest, since post-fire plant and bird communities are constrained by the original vegetation before the fire [23]. Burnt patch size was defined as the extent of the burnt area (ha) and was derived from digitalized maps from the Catalan Government.

### Data analyses

Generalized linear mixed models (GLMMs) with negative binomial error structure and log link function were used to analyse the effects of environmental and time since fire variables on vegetation cover as a means of assessing the importance of water deficit on post-fire plant regeneration (first prediction). The same model structure was used to analyse the influence of environmental, vegetation cover and time variables on the relative abundances of the four bird species and on bird species richness (second prediction). Given that the majority of transects were sampled in successive years, the results may be affected by temporal pseudoreplication [47]. Although birds and vegetation variables were sampled independently in successive years, topographic and climatic variables were unchanged over time. The inclusion of the calendar year as a Random factor could minimize this problem. Nevertheless, in order to avoid pseudoreplication completely, and to get similar sample size for time since fire categories, we selected a single sampling occasion (i.e. a census) for each transect. A stratified random selection was applied, trying to maximize the number of censuses of the worst represented time since fire categories (7 to 11 years after fire; S1 Fig). The dataset after the selection consisted of N = 694 bird counts and measures of environmental variables in the burnt areas.

Exploratory analyses showed quadratic relationships between explanatory variables and foliage cover (S1 Table), and linear relationships between explanatory and bird variables (S2 Table). Whenever a quadratic term was significant, it was used in subsequent models. We then combined environmental and temporal variables generating different biologically meaningful models (S3 Table). We also used the interaction between time since fire and water deficit to better assess whether aridity influences temporal patterns of vegetation and bird recovery. Transects, nested within locality nested within regional water deficit (WD_A_), were included as a Random factor in order to control possible site-based differences.

The structure of the minimum adequate model (MAM) was decided under the likelihood ratio (LR) test criterion; first by comparing models with different fixed effects we used maximum likelihood (ML) and second, we used restricted maximum likelihood (REML) to compute the estimates of coefficients for the MAMs achieved [48]. Normality and homoscedasticity were checked by visually inspecting the plots of residuals against fitted values. The most parsimonious models for each response variable were those whose Akaike information criterion (AIC) was within 2 units of the lowest AIC [49]. For these models, we calculated their conditional coefficient of determination considering fixed and random effect (R^2^_GLMM(c)_) and their AIC weight (relative likelihood of a model) [50, 51]. We then selected the most appropriate model following a criterion of less complexity, greater R^2^_GLMM(c)_ and greater AIC weight (S4 Table). To perform these analyses, we used the statistical package R [52], the lme4 package for GLMM [53] and the MuMIn package for AIC weight and R^2^_GLMM(c)_ [54].

Finally, we used the complete dataset (N = 3071 bird counts and measures of environmental variables) to obtain sufficient sample size to graphically represent the temporal evolution of the post-fire occurrence of the four bird species for the different water deficit categories. With this aim, the proportion of occurrence of shrubland birds in censuses was used, instead of relative abundance, because it provides a better comparison between species, thus minimizing the existing differences in numbers among them. Relative abundance was first transformed into presence (1) or absence (0) in each transect and then the proportion of occurrence of species was computed for all combinations of year since fire with each category of water deficit in the transects (WD_T_), using the seven categories previously defined for WD_A_. We removed transects with unburnt or lightly burnt patches to avoid their potential refuge role for birds in recently burnt areas. We finally eliminated transects with WD_T_ = 7, due to a low sample size, and grouped transects sampled seven to eleven years after fire into two categories of time since fire (7-8-9 years and 10–11 years), for the same reason.

## Results

Results are presented using the best model selected for each response variable according to our selection criteria (see Data analyses). The differences between two or more parsimonious models are usually small, and sometimes these models only differ by one or two variables (S4 Table)

### Vegetation recovery after fire along the water deficit gradient

Time since fire affected foliage cover of the three vegetation height layers, indicating the short term regeneration of vegetation after severe fires. GLMMs show quadratic negative temporal relationships with the lower (0 to 25 cm) and intermediate (25 to 100 cm) layers but a linear positive relationship with the upper layer (>100 cm). Moreover, water deficit also influenced the three vegetation layers, with arid areas having less foliage cover than wetter areas. However, our initial hypothesis stating that plant recovery should be quicker in the wettest areas was only supported for the upper vegetation layer; i.e. the interaction of time since fire and water deficit was selected only for the upper layer (Table 2).

**Table 2:** Summary of the variables of the generalized linear mixed models (GLMMs) analysing the influence of environmental and time since fire variables on foliage cover. Shaded cells represent unconsidered variables or interactions (see S1 Table and under *Methods*). Transect, nested within locality nested within regional water deficit (WD_A_), was used as Random factor.

Variable	Foliage cover (0–25 cm height layer)		Foliage cover (25–100 cm height layer)		Foliage cover (>100 cm height layer)	
	*b*±*SE*	*P*	*b*±*SE*	*P*	*b*±*SE*	*P*
**TSF**	7.87±1.19	< 0.01	13.68 ±1.31	< 0.01	3.46±0.49	< 0.01
**TSF^2^**	-0.55±0.10	< 0.01	-0.76±0.11	< 0.01		
**WD_T_**	-0.04±0.01	< 0.01	-0.03±0.01	0.04	-0.004±0.009	0.62
TSF*WD_T_					-0.006±0.002	< 0.01
**LOGGING**	-0.84±0.62	0.21	1.03±0.56	0.09	-0.5±0.42	0.26
**DEBRIS**	1.21±0.51	0.04	0.82±0.68	0.26	0.06±0.35	0.88
**PATCHES**					3.31±0.48	< 0.01
**HABITAT**			8.21±3.24	0.03	3.46±1.74	0.07
**AREA**					-0.002±0.0008	0.03

Slope (b) ± standard error (SE) and P-values (P) are shown for each relationship. TSF = time since fire (years); WD_**T**_ = water deficit (ml); LOGGING = extension of salvage logging (0–4); DEBRIS = presence of plant debris (0–8); PATCHES = extension of unburnt patches (0–4); HABITAT = type of pre-fire habitat and AREA = burnt area (ha).

**Fig 2:**
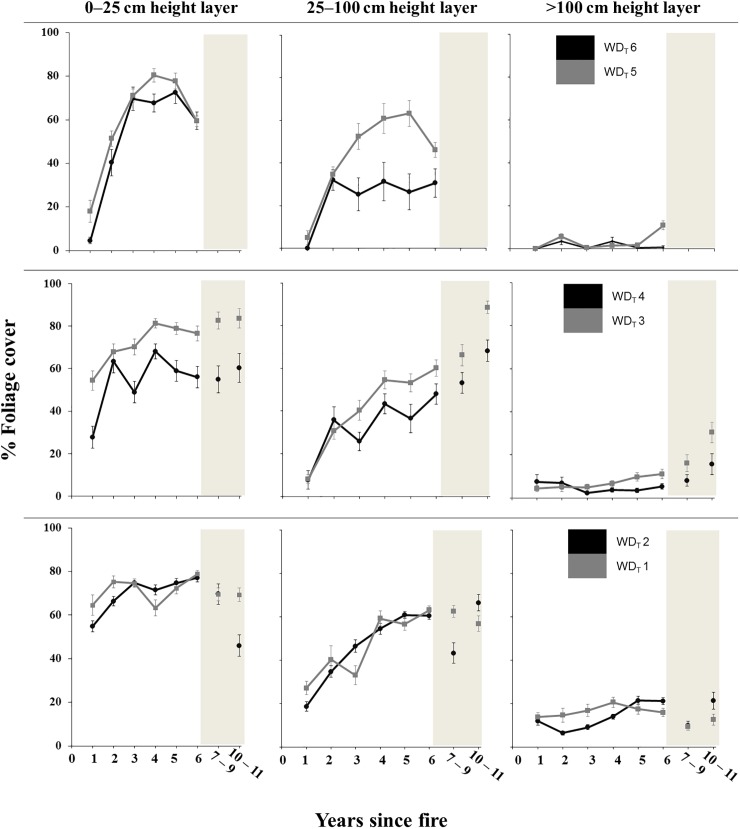
Variation in the percentage foliage cover explained by time since fire and water deficit. Relationships of the percentage foliage cover at three vegetation height layers (0–25 cm; 25–100 cm and >100 cm) with time since fire separated into categories of water deficit affecting the transect (WD_T_), using data from the entire database (N = 3071). Dots in the graphic are category means and error bars are standard errors.

Foliage cover showed different regeneration patterns depending on the specific vegetation height layer and on the water deficit category (Fig 2). Generally, foliage cover was higher in the wettest (WD_T_ 3–4 and especially WD_T_ 1–2) than in the driest areas in the early years after fire, and this was especially noticeable for the lowest vegetation layer and for the first year. Foliage cover in the lower and intermediate layers showed mostly unimodal temporal trends in the driest areas (WD_T_ 5–6) whereas trends were basically linear positive in the rest of water deficit categories. Finally, the upper layer foliage cover, which corresponds to long-stemmed shrubs and trees, was almost non-existent in the most arid areas for six years after fire (sampling size being insufficient thereafter). In other areas it could reach 30% of cover after eleven years.

Regarding management variables, the presence of timber harvest debris was positively related to the foliage cover of the three vegetation height layers. Logging of burnt forests had an uneven effect on foliage cover, with the lower and upper layers affected negatively and the intermediate layer affected positively. In addition, foliage cover was also influenced by fire characteristics, with the presence of unburnt vegetation patches positively influencing foliage cover of the upper layer. Burnt area size was negatively associated with the cover of the upper layer. Lastly, pre-fire habitat type was related to intermediate and upper layers (Table 2).

### Relationships among bird abundance and environmental variables

The species richness of the overall bird community in recently burnt areas had a positive linear relationship with time since fire, foliage cover for lower (0 to 25 cm) and upper (>100 cm) vegetation height layers and unburnt or lightly burnt patches, and a negative linear relationship with water deficit (Table 3). Although time since fire was positively associated with the relative abundances of the four focal bird species, water deficit also seems to be an important factor for two of them. Specifically, increased water deficit affected not only the relative abundances of *H*. *polyglotta* and *S*. *cantillans*, but also their recovery over the eleven years after the fire, as inferred from the negative interaction with time since fire, in concordance with our initial hypothesis. The *S*. *undata* and *S*. *melanocephala* populations, in contrast, did not show clear effects of aridity (Table 3).

**Table 3:** Summary of the variables of the generalized linear mixed models (GLMMs) analysing the influence of environmental and time since fire variables on overall bird richness and relative abundance of shrubland birds. Transect, nested within locality nested within regional water deficit (WD_A_), was used as Random factor.

Variable	Bird richness		*Hippolais polyglotta*		*Sylviacantillans*		*Sylviamelanocephala*		*Sylvia undata*	
	*b*±*SE*	*P*	*b*±*SE*	*P*	*b*±*SE*	*P*	*b*±*SE*	*P*	*b*±*SE*	*P*
**TSF**	0.51±0.07	< 0.01	0.24±0.03	< 0.01	0.13±0.04	0.02	0.71±0.06	< 0.01	0.13±0.02	< 0.01
**WD**_**T**_	-0.006±0.002	0.02	0.001±0.0007	0.05	-0.0002±0.0009	0.81				
TSF*WD_**T**_			-0.0006±0.0001	< 0.01	-0.0002±0.0001	0.13				
**C025**	0.01±0.006	0.08					0.01±0.004	0.01	-0.003±0.001	0.10
**C25100**			-0.003±0.002	0.13			-0.009±0.004	0.06	0.004±0.001	0.03
**C100**	0.03±0.008	< 0.01	0.02±0.003	< 0.01	0.01±0.003	< 0.01	0.02 ±0.006	< 0.01		
**LOGGING**			0.09±0.03	0.02			-0.01±0.07	0.87		
**DEBRIS**			0.04±0.03	0.19			0.18±0.06	0.01		
**PATCHES**	0.38±0.11	< 0.01	-0.08±0.04	0.05					-0.08±0.03	0.01
**HABITAT**					0.35±0.20	0.12				
**AREA**										

Slope (b) ± standard error (SE) and P-values (P) are shown for each relationship. TSF = time since fire (years); WD_T_ = water deficit (ml); C025, C25100 and C100 = foliage cover (%) for vegetation height layers 0–25 cm, 25–100 cm and >100 cm; LOGGING = extension of salvage logging (0–4); DEBRIS = presence of plant debris (0–8); PATCHES = extension of unburnt patches (0–4); HABITAT = type of pre-fire habitat and AREA = burnt area (ha).

**Fig 3:**
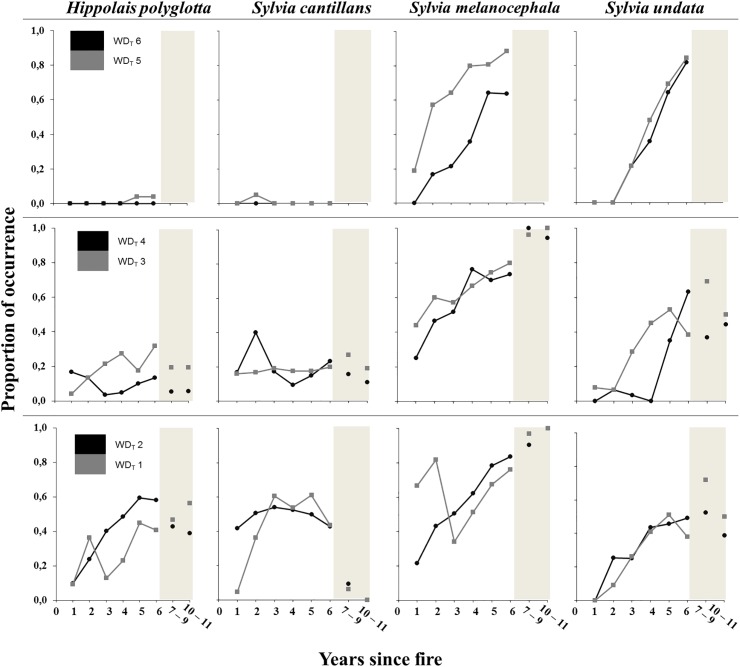
Variation in shrubland birds explained by time since fire and water deficit. Relationships of the proportion of occurrence (0 to 1) of the four shrubland birds with time since fire, separated into categories of water deficit affecting the transect (WD_T_), using data from the entire database (N = 3071).

Fig 3 shows that *H*. *polyglotta* was absent from the most arid areas (WD_T_ 5 and 6), and its recovery appeared to be quicker in wetter burnt areas (WD_T_ 1 and 2) than in those areas with intermediate water deficit (WD_T_ 3 and 4). *S*. *cantillans* shows a temporal pattern of occurrence similar to *H*. *polyglotta*, although it followed a unimodal trend, with very low proportion of occurrence from seven years after fire. The occurrence of *S*. *melanocephala* in the first year after fire was highest in mesic areas and lowest in the driest areas. Six years later, the differences among areas with different water deficit categories tended to disappear and the proportion of occurrence of the species was high, at around 0.7 to 0.9. Finally *S*. *undata* was the only species absent during the first year after fire. In the driest areas (WD_T_ 5 and 6) *S*. *undata* rapidly increased following wildfire and attained its highest occurrence six years after fire.

Finally, the additional factors of vegetation structure, fire characteristics and post-fire management practices were associated with changes in the occurrence of the four focal warbler species. *H*. *polyglotta* relative abundance exhibited a positive linear relationship with foliage cover at the upper vegetation height layer, salvage logging and presence of plant debris, but negative relationships with foliage cover at the intermediate (25 to 100 cm) vegetation height layer and with unburnt or lightly burnt patches. *S*. *cantillans* showed positive relationships with foliage cover at the upper vegetation layer and type of habitat, being more abundant in wooded than in unwooded areas. *S*. *melanocephala* showed linear positive relationships with foliage cover at both lower and upper vegetation layers and presence of plant debris but negative relationships with cover at the intermediate vegetation layer and with salvage logging. Finally, *S*. *undata* showed positive relationships with foliage cover at the intermediate vegetation height layer, but negative with cover at the lower vegetation layer and unburnt or lightly burnt patches (Table 3).

## Discussion

The contemporary modification of fire regimes, through the changes in human land use and climate [55], influences biodiversity at different scales [56]. An increase in water deficit, as predicted for the Mediterranean Basin, could even hinder the recovery of vegetation and fauna after wildfire. Our results show that both foliage cover and bird species relative abundance were associated to time since fire in all models generated. Nevertheless, water deficit also appeared as a predictor for six of the eight response variables studied. Specifically, it had an effect on foliage cover of the three vegetation height layers, on bird species richness, and on *H*. *polyglotta*, and *S*. *cantillans* numbers. Furthermore, in agreement with our hypotheses, aridity hampered the recovery of the upper vegetation layer and of *H*. *polyglotta* and *S*. *cantillans* populations.

### Vegetation recovery after fire across the water deficit gradient

Our main question regarding vegetation was to assess whether aridity affected plant recovery across an aridity gradient, and our results showed different effects of water deficit throughout post-fire succession. Mediterranean vegetation starts to regenerate shortly after the disturbance, both in areas of high and low aridity, although there was a trend of greater foliage cover in wettest areas after fire. The results obtained support our initial hypothesis for the upper vegetation height layer (>100 cm). In the time frame of our study, the lower (0–25 cm) and intermediate layers (25–100 cm) can reach considerable cover irrespective of aridity, but this is not the case of the upper layer. This fact may be related to the different plant strategies dominating the sites before the fire. Seeders, that regenerate by seedlings from fire-protected seeds stored in the soil or in the canopy bank [57], are more frequent in dry areas whereas sprouters, resprouting from fire resistant structures [58], tend to dominate in wettest areas [59]. Therefore, in addition to contrasting water availability, the rapid regeneration [60] and the larger size of sprouters also explains why aridity affected the recovery of the upper layer.

Our results therefore show that aridity affects the post-fire establishment and structure of the vegetation, although each vegetation height layer shows particular dynamics in relation to water deficit and time since fire (Fig 2). In semiarid areas, plant species richness may correlate well with rainfall during the first five years after fire [61]. Moreover, the presence of plant debris–either scattered or stacked in piles on the ground after logging–seemed to assist the regeneration of the lower and intermediate layers of vegetation in a number of ways: it may provide protection to plants and seeds from herbivores [62]; it can create microclimates favourable to plant growth and development [63]; it can increase the number of seeds in the soil due to deposition by birds that use such structures as perches [25] and soil depth and nutrient concentration may increase around such debris as it retains runoff [64]. Furthermore, the presence of unburnt or partially burnt areas contributed to greater foliage cover of the upper layer because tall shrubs and trees are maintained in these patches. These areas can play a decisive role as refuges for fauna that has fled the burnt area and as sources of future colonisers [65]. Finally, the negative relationship found between the size of the burnt area and the cover of the upper vegetation layer could result from the lower fire severity usually found in small burnt areas [66], where more foliage can remain in the canopy and subcanopy.

### Relationships of bird abundance with aridity and time since fire

Warblers gradually increased their populations in line with the increase in shrub cover in the burnt areas we studied, but showed different temporal dynamics depending on the habitat structure requirements of each species. Moreover, overall bird species richness increased with time since fire and the presence of unburnt or lightly burnt patches. These patches may act as habitat islands for several species [65] and can contribute to landscape heterogeneity allowing the coexistence of bird species with different habitat requirements [26]. Bird species richness also showed a slight negative effect of aridity that can be related to less complex habitat structure in arid areas. Climate change in the Mediterranean will likely increase water deficit and lead to greater water stress on Mediterranean vegetation [7]. A geographical aridity gradient provides a proxy for the pressure that animals experience with increasing aridity, including decreasing water supply and food availability and increasing air temperatures [67]. In a climate change context, our results point towards an effect of increasing aridity on the speed of vegetation recovery after fire, likely slower in the future. The bird species richness and the abundance of shrubland birds would therefore be negatively affected during the first years after a wildfire.

*Sylvia* and *Hippolais* warblers showed habitat preferences and temporal trends that were, in general, in accordance with their known requirements [40]. Moreover, the speed of the post-fire recovery of *H*. *polyglotta* and *S*. *cantillans* was affected by aridity. *H*. *polyglotta* prefers the wettest areas with bramble thickets and lush shrublands, although it was scarce in the most humid of the study areas due to altitude constraints, this species being uncommon above 1000 m [68]. Hence, in more arid habitats the species is confined–along with other birds, such as the nightingale (*Luscinia megarhynchos*) and the Eurasian wren (*Troglodytes troglodytes*)–to the vicinity of waterways, as water availability favours a rapid recovery of vegetation [26]. *H*. *polyglotta* also preferred logged areas, perhaps reflecting a positive association between logging occurrence and rainfall, since humid burnt forests seem to be more frequently logged than arid ones. On the other hand, *S*. *cantillans*, usually occurs in shrublands with scattered small trees or high shrubs [69], and this explains its positive association with wooded habitats in our models. The effect of aridity on this species was also consistent with the hypotheses of the study, since its recovery was much slower in arid areas and gradually increased its abundance with the regeneration of the upper vegetation layer. Since *S*. *undata* prefers dense scrubland, it was not frequently observed in burned areas until the intermediate vegetation layer had recovered. Finally, *S*. *melanocephala* relative abundance increased with increasing foliage cover and presence of plant debris [26]. Both species appeared unaffected by aridity at the spatial scale of our study. They are widespread in Catalonia, independently of the geographical differences in rainfall [70].

Overall, the presence of Mediterranean warblers after wildfire is determined by the recovery of the shrub layer, which in turn is influenced by both time since fire and the dryness associated with temperature, rainfall and altitude. The effect of aridity on birds appears to be indirectly modulated through changes in the vegetation structure, because water deficit alone, apart from the most arid environments, is unlikely to directly affect the occurrence of insectivore birds [16]. Our results show that time since fire is an important variable in all the models we generated to explain both bird species relative abundances and foliage cover in burnt Mediterranean areas. The importance of time since fire allows us to examine potential delays in bird recolonization in arid areas, although such a delay was only noticed for *S*. *undata* (Fig 3). Quadratic temporal patterns were found for the lower and intermediate vegetation layers, but not for bird species richness and relative abundances, likely due to the duration of the study. It is also worth mentioning that the presence of plant debris, mostly derived from salvage logging, both scattered and piled, appeared as an explanatory factor for *H*. *polyglotta* and *S*. *melanocephala*. Piles of burnt wood have been shown to be particularly important for *Sylvia* warblers in the first years following the fire [26]. However, the presence of significant cover, either having survived the fire or regenerated shortly after it, may reduce the beneficial effect of logging remnants.

### Aridity and conservation in fire-prone areas

Wildfires are usually considered as natural disturbances that can determine habitat structure and food resources, and increase biological diversity. Fire regimes have promoted adaptive mechanisms in plants of the Mediterranean Basin [71]. However, recent studies suggest that current and future changes in fire regimes can be detrimental to plant communities [72], produce significant losses of soil [73] and negatively affect *Sylvia* warblers [74] over large areas. At the same time, water deficit has increased in many areas, especially in Mediterranean-climate regions, due to temperature increases [5] and this may affect shrub cover and bird populations that depend on shrublands. Future scenarios therefore make it necessary to improve our knowledge on how aridity affects post-fire processes, especially in areas near the extremes of the aridity gradient. This information can be used to determine suitable actions to minimize the impact of aridity in burnt areas, for example by maximizing the recolonization of seed dispersers, such as warblers, to help restore plant communities. Building wood debris in recently burnt and logged forests or modifying fire frequency combining wildfire prevention and prescribed burning are possible strategies to achieve these goals.

## Supporting information

S1 Appendix Thornthwaite equation. Details of the Thornthwaite equation used to calculate the water deficit gradient.
(DOCX)

S1 Fig Data selection. Selection of transects for analyses.
(DOCX)

S1 Table Influence of time since fire and water deficit on foliage cover. Summary of generalized linear mixed models (GLMMs) analysing the influence of time since fire and its quadratic term, and water deficit and its quadratic term, on the foliage cover of three vegetation layers.
(DOCX)

S2 Table Influence of time since fire and water deficit on bird species richness and shrubland bird relative abundances. Summary of GLMMs analysing the influence of time since fire and its quadratic term, and water deficit and its quadratic term, on bird species richness, and on the relative abundances of *Hippolais polyglotta*, *Sylvia cantillans*, *Sylvia melanocephala* and *Sylvia undata*.
(DOCX)

S3 Table Environmental variables used in the analyses of bird variables. Combinations of environmental variables used in the GLMMs analysing bird species richness and warbler relative abundances.
(DOCX)

S4 Table Selection of best models. Models generated for each response variable (vegetation height layers, bird species richness and relative abundance of warbler species), whose Akaike information criterion (AIC) was within 2 units of the lowest AIC.
(DOCX)
