# Supplementary material for: Aridity influences the recovery of vegetation and shrubland birds after wildfire
Source: PLoS One. 2017 Mar 29;12(3):e0173599. doi: 10.1371/journal.pone.0173599 (PMC5371301; doi:10.1371/journal.pone.0173599)
Supplement: S1 Appendix — Details of the Thornthwaite equation used to calculate the water deficit gradient. (DOCX) [file pone.0173599.s001.docx]

**S1 Appendix.** Thornthwaite equation (Thornthwaite 1948, Black 2007): $WD = PET - ETr$.

Where $PET=16\left( \frac{L}{12} \right)\left( \frac{N}{30} \right)\left( \frac{10T_{a}}{I} \right)^{\alpha}$ is the estimated potential evapotranspiration (mm/month); $L$ is the average day length (hours) of the month being calculated; $N$ is the number of days in the month; $T_{a}$is the average daily temperature (degrees Celsius; if this is negative, 0 is used) of the month being calculated; $I=\sum_{i=1}^{12} \left( \frac{T_{ai}}{5} \right)^{1.514}$ is a heat index which depends on the 12 monthly mean temperatures; $\alpha=\left( 6.75\times{10}^{-7} \right)I^{3}-\left( 7.71\times{10}^{-5} \right)I^{2}+\left( 1.792\times{10}^{-2} \right)I+ 0.49239$.

$ETr=\sum_{i-1}^{12} \left( P_{ai}-{\Delta S}_{ai} \right)$ is the estimated real evapotranspiration (mm/month). The last equation balances the change in water stored within the basin (S) with inputs and exports; $P_{a}$ is the average daily precipitation (mm) of the month being calculated; ${\Delta S}_{a}=(S_{a}-S_{a-1})$ is the change in available water storage; and $S_{a}=(S_{a-1}+(P_{a}-{PET}_{a}))$ is the useful water storage for a given month.
