# Supplementary material for: Aridity influences the recovery of vegetation and shrubland birds after wildfire
Source: PLoS One. 2017 Mar 29;12(3):e0173599. doi: 10.1371/journal.pone.0173599 (PMC5371301; doi:10.1371/journal.pone.0173599)
Supplement: S1 Fig — Selection of transects for analyses. (DOCX) [file pone.0173599.s002.docx]

**
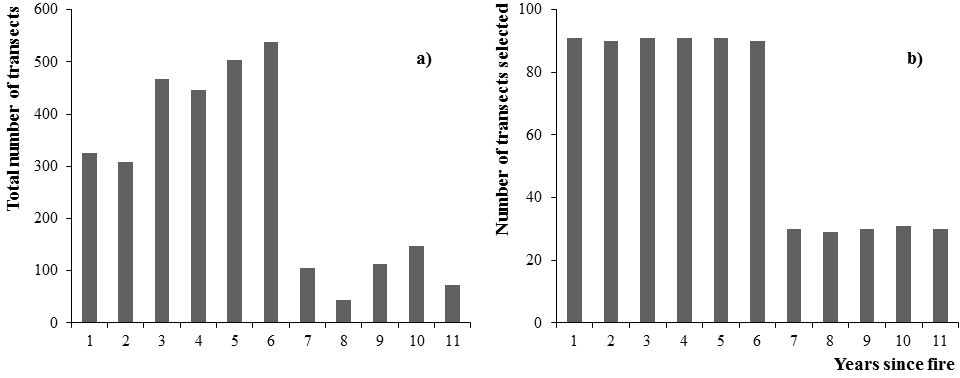
**

**S1 Fig**. Selection of transects for analyses, where (a) shows the total number of bird-vegetation transects (N_Total_ = 3071) in relation to time since fire (most transects were sampled over several years) and (b) shows the number of transects selected (N_Selected_ = 694) for analyses, after choosing a single census occasion per transect and with the aim of maximizing the number of samples of the worst represented time since fire categories (7 to 11 years since fire).
