# Supplementary material for: Aridity influences the recovery of vegetation and shrubland birds after wildfire
Source: PLoS One. 2017 Mar 29;12(3):e0173599. doi: 10.1371/journal.pone.0173599 (PMC5371301; doi:10.1371/journal.pone.0173599)
Supplement: S1 Table — Summary of generalized linear mixed models (GLMMs) analysing the influence of time since fire and its quadratic term, and water deficit and its quadratic term, on the foliage cover of three vegetation layers. (DOCX) [file pone.0173599.s003.docx]

**S1 Table.** Summary of generalized linear mixed models (GLMMs) analysing the influence of time since fire (TSF) and its quadratic term (TSF^2^), and water deficit (WD_T_) and its quadratic term (WD_T_^2^), on the three variables of habitat structure (foliage covers of three vegetation layers).

| **Variable** | **Foliage cover**  **(0-25 cm height layer)** | | **Foliage cover**  **(25-100 cm height layer)** | | **Foliage cover**  **(>100 cm height layer)** | |
| --- | --- | --- | --- | --- | --- | --- |
|  | *b±SE* | *P* | *b±SE* | *P* | *b±SE* | *P* |
| **TSF** | **7.66±1.15** | **< 0.01** | **13.95±1.27** | **< 0.01** | 1.27±0.82 | 0.17 |
| **TSF^2^** | **-0.55±0.10** | **< 0.01** | **-0.81±0.11** | **< 0.01** |  | (0.36) |
| **WD_T_** | -0.04±0.01 | 0.12 | -0.04±0.01 | 0.01 | -0.03±0.006 | 0.06 |
| **WD_T_^2^** |  | (0.92) |  | (0.15) |  | (0.9) |

Slope (b) ± standard error (SE) and P-values (P) are shown for each relationship.

Unimportant (P ≥ 0.01) quadratic terms were excluded from the models and P-values of the removed terms are shown in parentheses.

Bold models are those whose P<0.01.
