# Supplementary material for: Aridity influences the recovery of vegetation and shrubland birds after wildfire
Source: PLoS One. 2017 Mar 29;12(3):e0173599. doi: 10.1371/journal.pone.0173599 (PMC5371301; doi:10.1371/journal.pone.0173599)
Supplement: S2 Table — Summary of GLMMs analysing the influence of time since fire and its quadratic term, and water deficit and its quadratic term, on bird species richness, and on the relative abundances of Hippolais polyglotta, Sylvia cantillans, Sylvia melanocephala and Sylvia undata. (DOCX) [file pone.0173599.s004.docx]

**S2 Table.** Summary of generalized linear mixed models (GLMMs) analysing the influence of time since fire (TSF) and its quadratic term (TSF^2^), and water deficit (WD_T_) and its quadratic term (WD_T_^2^), on bird species richness, and on the relative abundances of *Hippolais polyglotta*, *Sylvia cantillans*, *Sylvia melanocephala* and *Sylvia undata*.

| **Variable** | **Species**  **richness** | | ***Hippolais***  ***polyglotta*** | | ***Sylvia***  ***cantillans*** | | ***Sylvia melanocephala*** | | ***Sylvia***  ***undata*** | |
| --- | --- | --- | --- | --- | --- | --- | --- | --- | --- | --- |
|  | *b±SE* | *P* | *b±SE* | *P* | *b±SE* | *P* | *b±SE* | *P* | *b±SE* | *P* |
| **TSF** | 0.62±0.07 | **< 0.01** | **0.17±0.02** | **< 0.01** | 0.09±0.02 | 0.01 | 0.72±0.05 | **< 0.01** | 0.14±0.02 | **< 0.01** |
| **TSF^2^** |  | (0.50) |  | (0.02) |  | (0.08) |  | (0.3) |  | (0.72) |
| **WD_T_** | -0.008±0.002 | **< 0.01** | -0.001±0.0005 | 0.29 | -0.001±0.0006 | 0.06 | -0.004±0.002 | 0.97 | -0.0004±0.0003 | 0.27 |
| **WD_T_^2^** |  | (0.52) |  | (0.86) |  | (0.28) |  | (0.69) |  | (0.33) |

Slope (b) ± standard error (SE) and P-values (P) are shown for each relationship.

Unimportant (P ≥ 0.01) quadratic terms were excluded from the models and P-values of the removed terms are shown in parentheses.

Bold models are those whose P<0.01.
