# Supplementary material for: Aridity influences the recovery of vegetation and shrubland birds after wildfire
Source: PLoS One. 2017 Mar 29;12(3):e0173599. doi: 10.1371/journal.pone.0173599 (PMC5371301; doi:10.1371/journal.pone.0173599)
Supplement: S3 Table — Combinations of environmental variables used in the GLMMs analysing bird species richness and warbler relative abundances. (DOCX) [file pone.0173599.s005.docx]

**S3 Table**. Summary of combinations of environmental variables used in the GLMMs analysing bird species richness and warbler relative abundances. Time since fire (TSF) is replaced by TSF + TSF^2^, if the quadratic term has been previously selected (Table S2). Similarly, the interaction TSF*WD_T_ was replaced by TSF^2^*WD_T_. “Vegetation layers” corresponds to seven different combinations of the three vegetation height layers (measured as % foliage cover).

| **Number** | **Combination of explanatory variables analysed** |
| --- | --- |
| 01 | TSF |
| 02 | TSF + Vegetation layers |
| 03 | TSF + HABITAT + AREA + Vegetation layers |
| 04 | TSF + HABITAT + Vegetation layers |
| 05 | TSF + PATCHES |
| 06 | TSF + PATCHES + Vegetation layers |
| 07 | TSF + PATCHES + HABITAT + AREA + Vegetation layers |
| 08 | TSF + PATCHES + HABITAT + Vegetation layers |
| 09 | TSF + LOGGING + DEBRIS |
| 10 | TSF + LOGGING + DEBRIS + Vegetation layers |
| 11 | TSF + LOGGING + DEBRIS + PATCHES |
| 12 | TSF + LOGGING + DEBRIS + PATCHES + Vegetation layers |
| 13 | TSF + LOGGING + DEBRIS + PATCHES + HABITAT + AREA + Vegetation layers |
| 14 | TSF + WD_T_ |
| 15 | TSF + WD_T_ + Vegetation layers |
| 16 | TSF + WD_T_ + HABITAT + AREA + Vegetation layers |
| 17 | TSF + WD_T_ + HABITAT + Vegetation layers |
| 18 | TSF + WD_T_ + PATCHES |
| 19 | TSF + WD_T_ + PATCHES + Vegetation layers |
| 20 | TSF + WD_T_ + PATCHES + HABITAT + AREA + Vegetation layers |
| 21 | TSF + WD_T_ + PATCHES + HABITAT + Vegetation layers |
| 22 | TSF + WD_T_ + LOGGING + DEBRIS |
| 23 | TSF + WD_T_ + LOGGING + DEBRIS + Vegetation layers |
| 24 | TSF + WD_T_ + LOGGING + DEBRIS + PATCHES |
| 25 | TSF + WD_T_ + LOGGING + DEBRIS + PATCHES + Vegetation layers |
| 26 | TSF + WD_T_ + LOGGING + DEBRIS + PATCHES + HABITAT + AREA + Vegetation layers |
| 27 | TSF + WD_T_ + TSF*WD_T_ |
| 28 | TSF + WD_T_ + TSF*WD_T_ + Vegetation layers |
| 29 | TSF + WD_T_ + TSF*WD_T_ + HABITAT + AREA + Vegetation layers |
| 30 | TSF + WD_T_ + TSF*WD_T_ + HABITAT + Vegetation layers |
| 31 | TSF + WD_T_ + TSF*WD_T_ + PATCHES |
| 32 | TSF + WD_T_ + TSF*WD_T_ + PATCHES + Vegetation layers |
| 33 | TSF + WD_T_ + TSF*WD_T_ + PATCHES + HABITAT + AREA + Vegetation layers |
| 34 | TSF + WD_T_ + TSF*WD_T_ + PATCHES + HABITAT + Vegetation layers |
| 35 | TSF + WD_T_ + TSF*WD_T_ + LOGGING + DEBRIS |
| 36 | TSF + WD_T_ + TSF*WD_T_ + LOGGING + DEBRIS + Vegetation layers |
| 37 | TSF + WD_T_ + TSF*WD_T_ + LOGGING + DEBRIS + PATCHES |
| 38 | TSF + WD_T_ + TSF*WD_T_ + LOGGING + DEBRIS + PATCHES + Vegetation layers |
| 39 | TSF + WD_T_ + TSF*WD_T_ + LOGGING + DEBRIS + PATCHES + HABITAT + AREA + Vegetation layers |
| 40 | WD_T_ |
| 41 | WD_T_+ Vegetation layers |
| 42 | WD_T_ + HABITAT + AREA + Vegetation layers |
| 43 | WD_T_ + HABITAT + Vegetation layers |
| 44 | WD_T_ + PATCHES |
| 45 | WD_T_ + PATCHES + Vegetation layers |
| 46 | WD_T_ + PATCHES + HABITAT + AREA + Vegetation layers |
| 47 | WD_T_ + PATCHES + HABITAT + Vegetation layers |
| 48 | WD_T_ + LOGGING + DEBRIS |
| 49 | WD_T_ + LOGGING + DEBRIS + Vegetation layers |
| 50 | WD_T_ + LOGGING + DEBRIS + PATCHES |
| 51 | WD_T_ + LOGGING + DEBRIS + PATCHES + Vegetation layers |
| 52 | WD_T_ + LOGGING + DEBRIS + PATCHES + HABITAT + AREA + Vegetation layers |
| 53 | Vegetation layers |
| 54 | HABITAT + AREA + Vegetation layers |
| 55 | HABITAT + Vegetation layers |
| 56 | PATCHES |
| 57 | PATCHES + Vegetation layers |
| 58 | PATCHES + HABITAT + AREA + Vegetation layers |
| 59 | PATCHES + HABITAT + Vegetation layers |
| 60 | LOGGING + DEBRIS |
| 61 | LOGGING + DEBRIS + Vegetation layers |
| 62 | LOGGING + DEBRIS + PATCHES |
| 63 | LOGGING + DEBRIS + PATCHES + Vegetation layers |
| 64 | LOGGING + DEBRIS + PATCHES + HABITAT + AREA + Vegetation layers |

TSF= time since fire (years); WD_T_= water deficit (ml); LOGING= extension of salvage logging; DEBRIS= presence of plant debris; PATCHES= extension of unburnt patches; HABITAT= type of pre-fire habitat and AREA = burnt area (ha).
