# Supplementary material for: Aridity influences the recovery of vegetation and shrubland birds after wildfire
Source: PLoS One. 2017 Mar 29;12(3):e0173599. doi: 10.1371/journal.pone.0173599 (PMC5371301; doi:10.1371/journal.pone.0173599)
Supplement: S4 Table — Models generated for each response variable (vegetation height layers, bird species richness and relative abundance of warbler species), whose Akaike information criterion (AIC) was within 2 units of the lowest AIC. (DOCX) [file pone.0173599.s006.docx]

**S4 Table.** Models generated for each response variable (vegetation height layers, bird species richness and relative abundance of warbler species), whose Akaike information criterion (AIC) were within 2 units of the lowest AIC.

|  | **Degrees of freedom** | **AIC** | **ΔAIC** | **AIC weight** | **R^2^_GLMM(c)_** | **Model structure** |
| --- | --- | --- | --- | --- | --- | --- |
| **Foliage cover**  **0 – 25 cm** | **9** | **6120.5** | **0.0** | **0.51** | **0.45** | **TSF + TSF2 + WD_T_ + LOGGING + DEBRIS** |
|  | 10 | 6121.8 | 1.3 | 0.26 | 0.46 | TSF + TSF2 + WD**_T_** + LOGGING + DEBRIS + PATCHES |
| **Foliage cover**  **25 – 100 cm** | **10** | **6255.5** | **0.0** | **0.38** | **0.47** | **TSF + TSF2 + WD_T_ + LOGGING + DEBRIS + HABITAT** |
|  | 11 | 6256.0 | 0.5 | 0.29 | 0.48 | TSF2*WD**_T_** + TSF + TSF2 + WD**_T_** + LOGGING + DEBRIS + HABITAT |
|  | 8 | 6257.1 | 1.6 | 0.18 | 0.46 | TSF + TSF2 + WD**_T_** + HABITAT |
| **Foliage cover**  **> 100 cm** | **12** | **5618.9** | **0.0** | **0.74** | **0.34** | **TSF*WD + TSF + WD_T_ + LOGGING + DEBRIS + PATCHES + HABITAT + AREA** |
| **Bird species**  **richness** | 11 | 3597.2 | 0.0 | 0.37 | 0.62 | TSF + WD_T_ + PATCHES + HABITAT + AREA + C025 + C100 |
|  | **9** | **3598.0** | **0.8** | **0.31** | **0.64** | **TSF + WD_T_ + PATCHES + C025 + C100** |
|  | 13 | 3598.6 | 1.4 | 0.20 | 0.62 | TSF + WD_T_ + LOGGING + DEBRIS + PATCHES + HABITAT + AREA + C025 + C100 |
|  | 10 | 3599.0 | 1.8 | 0.18 | 0.63 | TSF + WD_T_ + PATCHES + HABITAT + C025 + C100 |
| ***Hippolais***  ***polyglotta*** | **12** | **2070.6** | **0.0** | **0.27** | **0.56** | **TSF + WD_T_ +TSF*WD + LOGGING + DEBRIS + PATCHES + C25100 + C100** |
|  | 11 | 2071.1 | 0.5 | 0.26 | 0.44 | TSF + WD_T_ +TSF*WD + LOGGING + DEBRIS + PATCHES + C100 |
| ***Sylvia***  ***cantillans*** | **9** | **2183.7** | **0.0** | **0.48** | **0.42** | **TSF + WD_T_ +TSF*WD + C100 + HABITAT** |
|  | 8 | 2184.9 | 1.2 | 0.30 | 0.41 | TSF + WD_T_ + HABITAT + C100 |
|  | 8 | 2185.2 | 1.5 | 0.27 | 0.43 | TSF + WD_T_ + TSF*WD + C100 |
| ***Sylvia***  ***melanocephala*** | **10** | **3122.2** | **0** | **0.66** | **0.80** | **TSF + LOGGING + DEBRIS + C025 + C25100 + C100** |
|  | 11 | 3123.4 | 1.2 | 0.34 | 0.83 | TSF + WD_T_ + LOGGING + DEBRIS + C025 + C25100 + C100 |
| ***Sylvia***  ***undata*** | **8** | **1633.6** | **0.0** | **0.45** | **0.34** | **TSF + PATCHES + C025 + C25100** |
|  | 9 | 1634.2 | 0.6 | 0.32 | 0.35 | TSF + WD_T_ + PATCHES+ C025 + C25100 |
|  | 7 | 1635.3 | 1.7 | 0.23 | 0.30 | TSF + PATCHES + C25100 |

Bold models were those selected following a criterion of less complexity (degrees of freedom), greater R^2^_GLMM(c)_ and greater AIC weight.

Bird species richness= total number of bird species; TSF= time since fire (years); WD_T_= water deficit (ml); C025, C25100 and C100= foliage cover (%) for vegetation height layers 0-25 cm, 25-100 cm and >100 cm; LOGGING= extension of salvage logging; DEBRIS= presence of plant debris; PATCHES= extension of unburnt patches; HABITAT= type of pre-fire habitat and AREA= burnt area (ha).
